# Synergistic Enhanced Thermal Conductivity and Dielectric Constant of Epoxy Composites with Mesoporous Silica Coated Carbon Nanotube and Boron Nitride Nanosheet

**DOI:** 10.3390/ma14185251

**Published:** 2021-09-13

**Authors:** Yutao Hao, Qihan Li, Xianhai Pang, Bohong Gong, Chengmei Wei, Junwen Ren

**Affiliations:** 1 College of Electrical Engineering, Sichuan University, Chengdu 610065, China; 2018141441084@stu.scu.edu.cn (Y.H.); gongbohong@stu.scu.edu.cn (B.G.); weichengmei@stu.scu.edu.cn (C.W.); 2 College of Aviation Engineering, Civil Aviation Flight University of China, Guanghan 618307, China; cunzhangfangyang@163.com; 3 Security Management Center, State Grid Hebei Electric Power Research Institute, Shijiazhuang 050021, China; 2020223030004@stu.scu.edu.cn

**Keywords:** boron nitride nanosheet, multi-walled carbon nanotubes, composites, thermal conductivity, dielectric properties

## Abstract

Dielectric materials with high thermal conductivity and outstanding dielectric properties are highly desirable for advanced electronics. However, simultaneous integration of those superior properties for a material remains a daunting challenge. Here, a multifunctional epoxy composite is fulfilled by incorporation of boron nitride nanosheets (BNNSs) and mesoporous silica coated multi-walled carbon nanotubes (MWCNTs@mSiO_2_). Owing to the effective establishment of continuous thermal conductive network, the obtained BNNSs/MWCNTs@mSiO_2_/epoxy composite exhibits a high thermal conductivity of 0.68 W m^−1^ K^−1^, which is 187% higher than that of epoxy matrix. In addition, the introducing of mesoporous silica dielectric layer can screen charge movement to shut off leakage current between MWCNTs, which imparts BNNSs/MWCNTs@mSiO_2_/epoxy composite with high dielectric constant (8.10) and low dielectric loss (<0.01) simultaneously. It is believed that the BNNSs/MWCNTs@mSiO_2_/epoxy composites with admirable features have potential applications in modern electronics.

## 1. Introduction

Polymer dielectrics are extensively utilized in electronic devices because of their easy processing, flexibility, and light weight [1,2,3,4,5,6]. However, the low thermal conductivity of polymer dielectrics cannot meet the rising demand of efficient heat dissipation due to continues miniaturization electronic devices with high speed and high power [5,7,8,9]. Incorporation of thermally conductive fillers into polymer matrix is regarding as an effective strategy to improve the thermal conductivity of materials [10,11,12,13,14]. Among all of the thermally conductive fillers, boron nitride nanosheet (BNNS) is considered as the most promising filler due to its unique structure and ultrahigh thermal conductivity [15]. For instance, Chen et al. showed that a high thermal conductivity of 16.3 W m^−1^ K^−1^ can be obtained for the composite film by orderly arranging BNNSs using electrospinning technique. In addition, the composite film simultaneously exhibited low dielectric loss, high resistivity, and higher breakdown strength [16]. Zhao et al. fabricated a thermally conductive epoxy composites by constructing thermal conductive network using BNNSs and boron nitride microspheres (BNMSs). The obtained BNNSs/BNMSs/epoxy composites showed an enhanced thermal conductivity of 1.15 W m^−1^ K^−1^ at 30 wt% filler loading, which is five times higher than that of pure epoxy [17]. 

In modern applications, the highly thermally conductive performance and high dielectric constants are both essential. However, the inherent low dielectric constant of BNNSs, usually results in lower dielectric constant of the composites. In order to address this issue, electrically conductive fillers (such as multi-walled carbon nanotubes (MWCNTs), graphene, silicon carbide (SiC), etc.) are incorporated to improve the dielectric constant of the composite [18,19,20]. Especially, MWCNTs get more attention because of their high electrical conductivity and ultrahigh aspect ratio [21,22]. Unfortunately, although the addition of MWCNTs greatly improves the dielectric constant of the composites, it is also accompanied by a higher dielectric loss. Functionalization the surface of MWCNTs with dielectric layer is an effective strategy to restrain the increase of dielectric loss [10,23,24,25,26,27]. For instance, Yang et al. coated MWCNTs with a layer of polypyrrole, which contributed to a high dielectric constant and low dielectric loss for the composites. [28]

On the other hand, the combination of different fillers was considered as a promising strategy to increase the performance of composites. Liu et al. successfully adsorbed barium titanate nanoparticles on the surface of MWCNTs by sol–gel method, then grafted hyperbranched aromatic polyamide from the surface of barium titanate. Using the hybrid nanoparticle as filler, a dielectric loss of 0.28 and a high dielectric constant of 110 was achieved for the composite [29]. Wang et al. found that by combination of BNNSs and diethylenetriamine functionalized MWCNTs, low dielectric loss, and ultrahigh thermal conductivity can be achieved for the composite [30]. Thus, it is believed that high thermal conductivity and high dielectric constant can be simultaneously achieved by combinations of various types of fillers. In this study, we present a facile method to prepare multifunctional epoxy composites by combination of BNNSs and mesoporous silica layer coated MWCNT_S_ (MWCNT_S_@mSiO_2_) fillers. The MWCNT_S_@mSiO_2_ was prepared via a sol-gel method with the aid of cationic surfactant cetyltrimethyl ammonium bromide (CTAB). We systematically investigated the synergistic work of MWCNT_S_@mSiO_2_ and BNNSs on the dielectric and thermally conductive properties of composite. It is apparent that the BNNSs/MWCNTs@mSiO_2_/epoxy composites is a potential candidate for application in advanced electrics.

## 2. Experimental Section

### 2.1. Materials

The average particle size of h-BN was 200 nm, which was purchased from Aladdin Co., Ltd., China. Bisphenol A diglycidyl ether (E-51) with an epoxide equivalent weight of 192, methyl hexahydrophthalic anhydride (curing agent), and tris(dimethylaminomethyl)phenol (catalyst) were provided by Nantong Xingchen Synthetic Materials Co., Ltd., Nantong, China. Graphitized-OH functionalized MWCNTs, with an outer diameter of 30–50 nm and the average length of ~10 μm, were purchased from Chengdu Organic Chemistry Co., Ltd., Chinese Academy of Sciences, Chengdu, China. Sodium hydroxide (NaOH), CTAB and tetraethyl silicate (TEOS) were obtained from Shanghai Aladdin Biochemical, Shanghai, China. The other materials, such as deionized water (DI-H_2_O) and ethyl alcohol, were purchased from Sinopharm Chemical Reagent Beijing Co., Ltd., Beijing, China, and were used as received.

### 2.2. Fabrication of BNNSs, MWCNTs@mSiO_2_, and Epoxy Composites

As introduced in literature, we used the liquid exfoliation of h-BN via hydrothermal and ultrasonic treatments to prepare the BNNSs [17]. For example, 0.5 g h-BN was mixed with 100 mL isopropanol/DI H_2_O (*v*/*v* = 3/1) mixture. Then, sonicated the obtained dispersion at 40 kHz (200 W) for 2 h. The resulting dispersion was heated at 180 °C for 24 h after shift to a Teflon-lined stainless-steel autoclave. In order to eliminate the non-exfoliated h-BN, we centrifuged the resulting dispersions at 1000 rpm for 20 min after cooling. After decanting the supernatant, centrifuged it at the speed of 8000 rpm for 30 min to collect the exfoliated BNNSs. Finally, collected the resulting BNNSs after drying at 40 °C for 72 h under vacuum.

The MWCNTs@mSiO_2_ was synthesized by sol–gel methods [31,32]. Firstly, 0.5 g of MWCNTs, 0.25 g of CTAB, and 0.01 g of NaOH were added into 250 mL of DI H_2_O, and ultrasonically dispersed for 30 min to form a stable dispersion. Then, 25 mL of TEOS/ethyl alcohol (*v*/*v* = 1/4) solution was injected into the as-prepared MWCNTs dispersion. After shaking mildly for 5 min, the resulting mixture was heated at 60 °C without stirring for 10 h to coat silica on the surface of MWCNTs. After the coating processing, the product was washed at least 3 times by centrifugation (5000 rpm for 5 min) and redispersion in ethyl alcohol to eliminate unreacted organics and free silica particles. The remaining solids were dispersed in 250 mL of ethyl alcohol and heated for 5 h at 60 °C to remove the CTAB thoroughly. The resulting suspension was washed with ethyl alcohol after filtered through millipore filters (0.45 µm FG). The remaining solid was dried in a vacuum oven at 60 °C for 48 h.

**Figure 1:**
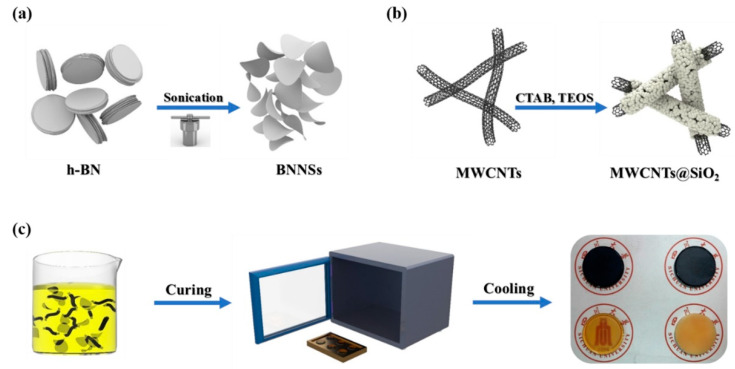
(**a**) The process of the liquid-phase exfoliation of h-BN. (**b**) Coating mesoporous silica on the surface of MWCNTs. (**c**) Preparation procedure of the BNNSs/MWCNTs@mSiO_2_/epoxy composites.

The composites were produced by solution blending method. Firstly, quantitative BNNSs and quantitative MWCNTs@mSiO_2_ were added into acetone solution, and dispersed the nanofillers by stirring at 350 rpm for 30 min. The epoxy, curing agent, catalyst were added into a separate beaker at a 100:80:1.6 weight ratio. Then the epoxy mixture and the required amount of BNNSs/MWCNTs@mSiO_2_ dispersion were mixed at the room temperature in order to prepare the BNNSs/MWCNTs@mSiO_2_/epoxy composites with different filling amounts (5%/0.01%, 5%/0.05%, 5%/0.10%, 10%/0.01%, 10%/0.05%, 10%/0.10%, 15%/0.01%, 15%/0.05%, 15%/0.10%, 15%/0.10%, 20%/0.01%, 20%/0.05%, 20%/0.10%). The resulting mixture was stirred at 350 rpm, 70 °C for 2 h in order to mixed evenly. The mixture was transferred to the preheated steel mold, degassing at 70 °C for 1 h and then 80 °C for 1 h to eliminate the residual bubbles. Finally, cured the composites at 120 °C for 2 h and 130 °C for another 2 h, respectively. The BNNSs/epoxy, MWCNTs@mSiO_2_/epoxy composites were prepared by the same way. The preparation processes of BNNSs, MWCNTs@mSiO_2_ and their epoxy composites are exposited in Figure 1.

### 2.3. Characterization 

The transmission electron microscopy (TEM, 200 kV, JEOL 2100F, Tokyo, Japan) and scanning electron microscopy (SEM, 5 kV, FEI, Waltham, MA, USA) were used to characterized the microstructure and morphology of MWCNTs@mSiO_2_, BNNSs, and the composites. The diffraction patterns of composites were recorded by the X-ray diffractometer (Philips X’Pert Pro MPD, Amsterdam, NLD) in the 2θ range (15–60°) at a scanning speed of 5°/min with the Cu Kα radiation (λ = 0.154 nm). A Horiba LabRAM HR Micro-Raman system (Kyoto, Japan) which equipped with a 633 nm laser excitation was used to get the Raman spectra from 100 to 2000 cm^−1^. A Nicolet 6700 spectrometer (Waltham, MA, USA) was used to record the Fourier transform infrared spectroscopy (FT-IR) from 400 to 4000 cm^−1^. The transient plane heat source method, (TPS2500s, Hot Disk AB, Uppsala, Sweden), was used to measure the thermal conductivity of composites at room temperature. The HP 4194A impedance analyzer was used to analyze the dielectric response of the composites in the frequency range of 10^2^−10^6^ Hz. The Keeithley 6517B (Tektronix, Beaverton, OR, USA) was used to test the volume resistivity of the samples. A dynamic mechanics analyzer (DMA, NETZSCH, DMA 242, Selb, Germany) was used to measure the dynamic thermomechanical properties of the samples. It was operating with a constant heating rate of 5 °C/min at 1 Hz. The composite papers surface temperature was recorded by the infra-red thermograph (FLIR T650sc, Wilsonville, OR, USA). A TGA 2950 thermogravimetric analyzer with TA instrument (Wilmington, NC, USA) was used to performed the Thermogravimetric analysis (TGA) of the composites at a N_2_ flowing rate of 20 mL/min and a heating rate of 10 °C/min.

## 3. Results and Discussion

**Figure 2:**
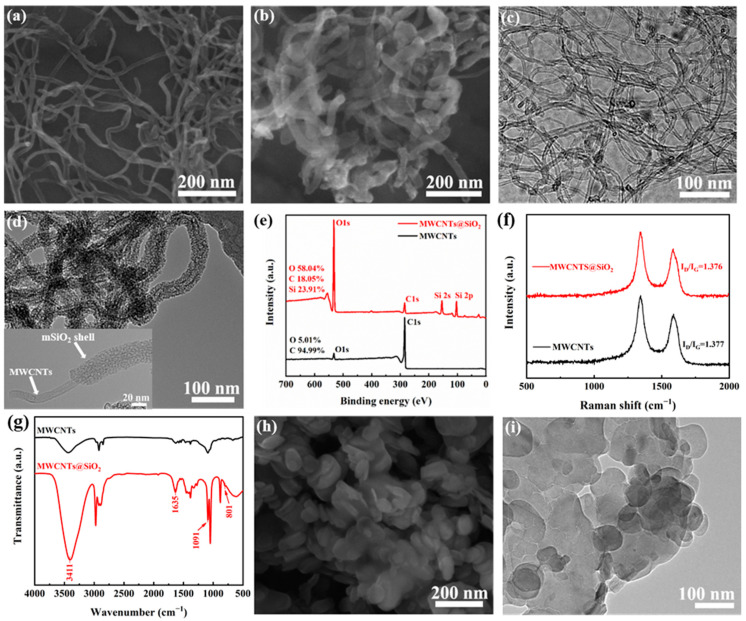
Characterization of MWCNTs and MWCNTs@mSiO_2_. SEM image of (**a**) MWCNTs and (**b**) MWCNTs@mSiO_2_. (**c**) MWCNTs. (**d**) TEM image of MWCNTs@mSiO_2_. Inset is an individual MWCNTs@mSiO_2_. (**e**) FT−IR spectra of MWCNTs and MWCNTs@mSiO_2_. (**f**) XPS general spectra of MWCNTs and MWCNTs@mSiO_2_. (**g**) Raman spectra of MWCNTs and MWCNTs@mSiO_2_. (**h**) SEM image of h-BN. (**i**) TEM image of BNNS.

Although MWCNTs possess excellent thermally conductive properties, high dielectric loss and the inferior electric insulation of the composites will be caused by its high electrical conductivity. In present study, we functionalized MWCNTs with mSiO_2_ dielectric layer to suppress the electrical conductivity of MWCNTs. As can be seen from the SEM images in Figure 2a,b, the MWCNTs@mSiO_2_ appear a thick diameter than that of MWCNTs. In addition, compared with the smooth surface of MWCNTs, the MWCNTs@mSiO_2_ exhibit a core–shell configuration, wherein the MWCNT is uniformly encapsulated by the mSiO_2_ layer as reveals in Figure 2c,d. The modification of mSiO_2_ layer can significantly improve the dispersibility of MWCNTs as well as enhance the interfacial interaction between epoxy matrix and MWCNTs.

The XRP analysis was used to evident the functionalization (Figure 2e). Comparison of the XPS wide spectra of neat MWCNTs and MWCNTs@mSiO_2_ revealed that Si 2s and Si 2p peaks were appeared at 154.08 eV and 103.08 eV, respectively, which indicating the success of surface modification of MWCNTs with mSiO_2_ layer [33]. In addition, the content of O element increase significantly from 5.01% of MWCNTs to 58.04% of MWCNTs@mSiO_2_. This increase also provide a compelling evidence for successfully functionalization of MWCNTs. The Raman spectra of MWCNTs and MWCNTs@mSiO_2_ are presented in the Figure 2f. The ratio of D- (∼1300 cm^−1^) and G-band (∼1580 cm^−1^) intensities (the I_D_/I_G_ ratio) exhibit a negligible change (from MWCNTs for 1.377 to 1.376 for MWCNTs@mSiO_2_). This suggests that the modification of mSiO_2_ does not cause additional C atomic lattice defects for MWCNTs, because the I_D_/I_G_ ratio is closely related to that of the hybridization of the carbon atoms in MWCNTs. Figure 2g exhibits the FT-IR spectra of MWCNTs and MWCNTs@mSiO_2_. The features of the symmetric stretching vibration peak of Si-O appears at 800 cm^−1^, and the anti-symmetric stretching vibration peak of Si-O-Si appears at 1090 cm^−1^. This also indicates that SiO_2_ are successfully coated on the surface of MWCNTs [34]. 

As shown in Figure 1a, the BNNSs were produced by ultrasonic exfoliation of h-BN assisting with the hydrothermal treatment. Because of the high-pressure and high-temperature of hydrothermal reaction, the interlayer interaction will weaken, and the interlayer distance will increase. As a result, the thick h-BN (Figure 2h) was exfoliated to a small and thin BNNSs (Figure 2i).

**Figure 3:**
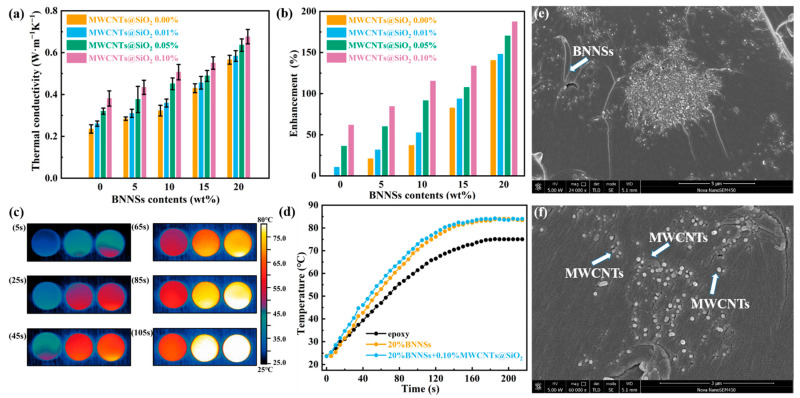
(**a**) Thermal conductivities of composites. (**b**) Enhancement efficiency of composites. (**c**) Infrared thermal imaging of composites; epoxy (left), BNNSs/epoxy (middle), BNNSs/MWCNTs@mSiO_2_/epoxy (right) (**d**) Curves of composites surface temperature as a function of time. (**e**,**f**) The cryogenically fractured surfaces morphologies SEM images of the BNNSs/MWCNTs@mSiO_2_/epoxy composites at different magnification.

The Figure 3a shows the variation of thermal conductivity of composite with the content of MWCNTs@mSiO_2_ and BNNSs. As expected, the thermal conductivity of the composite increases with the increase of filler content. It is worth noting that, the thermal conductivity improves slowly at the low filler contents. However, as the filling content is less than 15 wt%, with the increase of the filling content the thermal conductivity increases slightly (Figure 3b). When the filling content higher than 20 wt%, the thermal conductivity of the composite exhibits a remarkable increase of thermal conductivity. A high thermal conductivity of 0.68 W m^−1^ K^−1^ is achieved for composite at the filler contents of 20 wt%, which is 187% higher than that of pure epoxy. This typical variation of thermal conductivity can be explained by the network structure and distribution of the filler in the organic matrix. BNNSs is similar to islands in the matrix [35]. When the content of fillers is low, the high interfacial thermal resistance caused by the thick resin between fillers inhibits the formation of continuous thermal conduction path and prevents the diffusion of heat flow, and the thermal conductivity coefficient of the composites prepared increased slowly. When the filler content reaches a critical value, a “highway” which can be used as an efficient heat transfer will be established in the matrix. In this case, high thermal conductivity additives will dominate the thermal conductivity of composites, and will increase significantly with the increase of filler content.

In addition, the introduction of MWCNTs@mSiO_2_ can further improve the thermal conductivity of BNNSs/epoxy composites. The composite was heated from 23 °C to 85 °C sustainably to evaluate its heat absorption performance. Figure 3c,d shows the temperature distribution images and the corresponding Curves of composites surface temperature as a function of time. Among all the samples the BNNSs/MWCNTs@mSiO_2_/epoxy composites show the fastest increase and the highest peak of the surface temperature, showing its outstanding heat transfer efficiency. Those provide compelling evidence for the improvement of the thermal conductivity of epoxy composites by introducing the hybrid of BNNSs and MWCNTs@mSiO_2_. This phenomenon originates from the formation of the separated double heat conduction network. On the one hand, a thermal conductivity network has been established by BNNSs in the epoxy matrix. The contact between BNNSs and MWCNTs@mSiO_2_ greatly reduced the thermal resistance at the interface, resulting an improvement of the thermal conductivity of the composites. On the other hand, the addition of MWCNTs@mSiO_2_ established a new channel for phonon transmission. In this case, MWCNTs@mSiO_2_ mainly played the role of phonon transmission in coordination with BNNSs thermal conduction network (Figure 3e,f) [36]. As a result, a three dimensional thermally conductive network are generated in the composites, which contributes to the increase of the thermal conductivity. 

**Figure 4:**
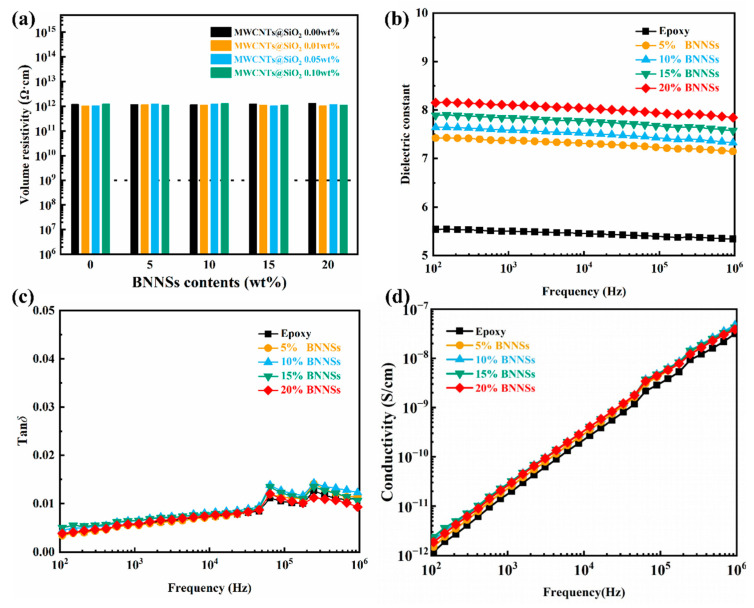
Electrical properties of the BNNSs/MWCNTs@mSiO_2_/epoxy composites. (**a**) Volume resistivity at different MWCNTs@mSiO_2_ contents. (**b**–**d**) Dielectric constants, dielectric loss, and AC conductivity at 0.1 wt% loading of MWCNTs@mSiO_2_ as a function of frequency.

In addition to the high thermal conductivity, exceptional dielectric properties are also important to utilize in electronic device. Figure 4a shows the volumetric resistivity of the MWCNTs@mSiO_2_/BNNSs/epoxy composite changes with the loading of BNNSs at different MWCNTs@mSiO_2_ contents. Owing to the electron transport path are obstructed by excellent electrical insulating of the BNNSs and mSiO_2_ layer, all the samples reveal a volume resistivity higher than 10^12^ Ω·cm. In terms of dielectric constant, the introduction of small amount of MWCNTs@mSiO_2_ could make a great change in the dielectric constant of the composite material. The dielectric constant of the pure epoxy at 10^3^ Hz is 5.50, the dielectric constant of composites increase to 8.10 with addition of 0.1 wt% MWCNTs@mSiO_2_ and 20 wt% BNNSs, which is about 47% higher than that of pure epoxy (Figure 4b). Furthermore, the dielectric constant of composites increase gradually with the increasing of BNNSs contents, which can be attributed to the introducing a lot of interfacial polarization. [28,37,38] The mini-capacitor principle can explain the increase of dielectric constant of composites. With the increase of filler content, many mini-capacitors with fillers as electrodes and epoxy matrix as dielectrics will be generated in the composites due to the decrease of the spacing between the nanoparticles. The dielectric constant of the composites will increase greatly because of those mini-capacitors. Moreover, the surface modification of mSiO_2_ not only inhibit the electrical conductivity of MWCNTs, but improve its dispensability as well, resulting in better dispersion of MWCNTs in the composites. Better dispersion leads to more small capacitances between MWCNTs and the polymer matrix, which leads to a significant increase in the dielectric constant of the composites. This also confirms the great effect of MWCNTs@mSiO_2_ on the improvement of the dielectric constant of composite materials. Similar results are reported in previous research, as summarized in recently review of Raghavan and coworkers [39]. More important, the dielectric losses of the composites remain low (<0.01). It shows that the addition of the MWCNTs@mSiO_2_ can improve dielectric constants of the composites without increasing the dielectric loss (Figure 4c). Dielectric loss is generally considered to consist of dipole loss, interfacial polarization loss, and conduction loss. For the composites filled with MWCNTs, the dielectric loss is mostly determined by the conduction loss. In previous reports, high dielectric losses were observed in MWCNTs composites due to its high leakage current. However, the dielectric loss of the composites maintaining at a low level, indicating that the mSiO_2_ dielectric layer can effective hinder the electrical connection between MWCNTs. This also can be evidenced by the negligible increase of electrical AC conductivity of composites as shown in Figure 4d.

**Figure 5:**
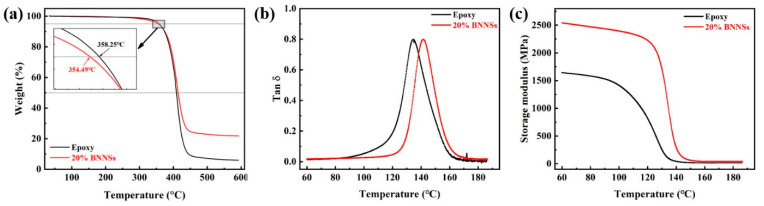
(**a**) TGA curves, (**b**) loss factor curves, and (**c**) storage modulus of the epoxy and BNNSs/MWCNTs@mSiO_2_/epoxy composite at 0.1 wt% loading of MWCNTs@mSiO_2_ as a function of temperature.

Figure 5a shows the TGA curves of epoxy and the composites. The decomposition temperature at a mass loss of 5% is defined as the initial thermal decomposition temperature. It can be observed that the initial decomposition temperature of epoxy resin is 358.25 °C, and the initial thermal decomposition temperature of BNNSs/MWCNTs@mSiO_2_/epoxy composites are 354.49 °C. This shows that the addition of BNNSs and MWCNTs@mSiO_2_ have little effect on the initial thermal decomposition temperature of epoxy matrix. The heat-resistance index of composites can be calculated according to previous paper. [40]

T_Heat-resistance index_ = 0.49 × [T_5_ + 0.6 × (T_30_ − T_5_)] (1)

The T_HRI_ is the heat-resistance index temperature. The T_5_ and T_30_ are the temperatures of decomposition which at 5 wt% and 30 wt% weight loss, respectively. As can be seen from Table 1. The T_HRI_ of composite was almost the same as that of pure epoxy, which indicate that the addition of MWCNTs@mSiO_2_, BNNSs, and epoxy has negligible effect on the heat resistance of epoxy resin. Moreover, as shown in Figure 5b, the composites exhibit a higher glass transition temperature (142 °C) than that of pure epoxy (135 °C). This can be attributed to the incorporation of BNNSs and MWCNTs@mSiO_2_ constrains the segmental movement of the epoxy chain and the reduction of the free volume. Furthermore, the storage modulus of the epoxy was significant increase by the incorporation of BNNSs and MWCNTs@mSiO_2_, which indicates the good reinforcement effect of BNNSs and MWCNTs@mSiO_2_ (Figure 5c). Although, for glass transition temperature, BNNS/MWCNTs@mSiO_2_ exhibit a lower enhancement efficiency than that of BNNS/BNMS. In terms of storage modulus, the hybrid of BNNS/MWCNTs@mSiO_2_ display much better enhancement effect than BNNS/BNMS [17].

## 4. Conclusions

In summary, a mesoporous SiO_2_ layer was used to modify the surface of MWCNTs to restrain the conductivity of MWCNTs. FT-IR, XPS, and Raman spectra showed that the MWCNTs was successfully coated by mesoporous SiO_2_ without destroying the crystal structures of MWCNTs. The BNNSs/MWCNTs@mSiO_2_/epoxy composite achieved a high thermal conductivity of 0.68 W m^−1^ K^−1^, which can be attributed to the effective establishment of thermally conductive networks due to the synergistic effect between BNNSs and MWCNTs@mSiO_2_. Furthermore, the electron transfer between MWCNTs can be impeded by the mesoporous SiO_2_ layer successfully, resulting in a high dielectric constant (8.10) and low dielectric loss (<0.01) of BNNSs/MWCNTs@mSiO_2_/epoxy composite. Moreover, the dynamic mechanical properties of the composites were improved due to the strong interfacial interaction between MWCNTs@mSiO_2_ and epoxy matrix. We believe that the findings of this study provide a facile approach to prepare materials with excellent dielectric properties and high thermal conductivity.

## Figures and Tables

**Table 1:** Characteristic thermal data of BNNSs/MWCNTs@mSiO_2_/epoxy nanocomposites.

Samples	Weight Loss Temperature/°C		T_HRI_/°C
	T_5_	T_30_	
Pure epoxy resin	358.25	397.75	187.16
0.1%MWCNTs@mSiO_2_/20%BNNSs/epoxy	354.49	400.96	187.36

## Data Availability

The data presented in this study are available on request from the corresponding author.
